# Retrospective cohort study on risk factors for development of gestational diabetes among mothers attending antenatal clinics in Nairobi County

**DOI:** 10.11604/pamj.2016.24.155.8093

**Published:** 2016-06-22

**Authors:** Maureen Atieno Adoyo, Charles Mbakaya, Venny Nyambati, Yeri Kombe

**Affiliations:** 1 Institute of Tropical Medicine and Infectious Diseases, Kenya; 2 Centre for Public Health Research, Kenya Medical Research Institute, Kenya; 3 College of Health Sciences, Department of Biochemistry, Jomo Kenyatta University of Agriculture and Technology, Kenya

**Keywords:** Retrospective cohort study, WHO, gestational diabetes

## Abstract

**Introduction:**

World Health Organization estimates that deaths resulting from diabetes will rise above 50% by the year 2020; hence urgent action is needed to reverse the trend notably through nutrition and lifestyle intervention among populations at risks. Studies have established that nutritional environment and physiology of the mother affects neonate's health at infancy and later in life thus this study sought to investigate the risk factors for development of gestational diabetes focusing age, weight, family history and pre-existing medical condition which could be modified to improve population health.

**Methods:**

A retrospective cohort study design was used. Subjects were sampled from selected maternity facilities in Nairobi and were subjected to oral glucose test to ascertain Gestational Diabetes mellitus (GDM) status. A questionnaire was administered to a sample of 238 respondents. Quantitative data was then analyzed as descriptive statistic, univariate and multivariate regression.

**Results:**

Average age for mothers with GDM was high with a mean of 33.06 (95% C.I: 31.59-34.52) compared to a mean of 27.9 (95% C.I: 27.01-28.78) for non-GDM mothers. Weight before pregnancy was high with mean of 74.04 (95% C.I: 70.82-77.30) among mothers with GDM compared to mean of 60.27 (95% C.I:58.59-61.96) among non-GDM mothers. Mothers with diabetic history in the family had twice the risk of developing GDM (OR= 2.27; 95% C.I: 1.23-4.17) compared to those who did not observe diabetic history in the family.

**Conclusion:**

Gestational diabetes cases are relatively high. Age advancement; high weight and diabetic history in family are determining factors for development of diabetes among pregnant women.

## Introduction

A WHO report indicates that burden of Non-Communicable Diseases (NCDs) is rising at an alarming rate among low Income Countries (LICs) imposing large, avoidable costs in socio-economic terms among population and communities. Further, diabetes case is projected to rise by more than 50% by the year 2020, if urgent interventions are not put in place to reverse the trend [1]. In Africa, the movement from a rural lifestyle to a more industrial urbanized way of life is mainly responsible for the evolving problem of chronic diseases such as diabetes and other NCDs [2]. Maternal factors including medical condition, weight, age and nutrition during development of fetus may influence future health and disease susceptibility such as adverse metabolic conditions including obesity, diabetes and cardiovascular disease [3] hence the need to have intervention at individual developmental stages among high risk population. Mayo Clinic suggests that any woman is at risk of developing gestational diabetes but some have a higher risk than others, women older than 25 years have increased risks, other factors such as family or personal history is also a determining factor to the development of GDM condition [4]. Further, increased GDM risk is determined by a woman's pre-diabetes condition; a precursor to type 2 diabetes and when a family member of a pregnant woman, such as a parent or sibling, has type two 2 diabetes. A woman who is overweight before pregnancy and had had very large baby in the previous deliveries or had a still birth is also thought to be at risk of developing gestational diabetes [4]. A significant proportion of pregnancies approximately 7% are complicated by GDM related to birth defects and also the leading causes of perinatal mortality across the world [5]. Generally, studies done in Africa related to GDM are few and scanty in information therefore, the need to conduct further research to explore risk factors that lead to GDM specific to developing Countries in Africa.

## Methods

A retrospective cohort study was conducted among mothers attending selected maternity facilities in Nairobi County; which comprises of six sub-counties and has a total of 92 maternity facilities put into different categories of: local Authority, Ministries of Health, Non-Governmental and Private enterprise based on institution ownership. An eligibility criterion for study participants was carefully determined by prescribed inclusion criteria before the commencement of the study.

**Inclusion criteria:** Mothers attending ANC in selected maternity facilities; mother who are on third trimester of pregnancy; willingness to take Glucose intolerant test; mothers willing to participate in the study by signing the consent form. A proportionate sample size determination formulae [6] was used to derive a definite sample for the two groups (GDM and non-GDM), effect size for each group was within 10% point of true difference, and 95% Confidence. Factoring in the effect size, the estimate of proportion of among mothers with GDM was assumed to be 23%.

n= {Z_1-α/2_√[2P(1-P)]+Z_1-β_√[P_1_(1- P_1_+P_2_(1- P_2_)]}^2^/(P_1_-P_2_)^2^

Where:

n=Minimum required sample size;

α = Type I error (0.05);

β= Type II error (0.10);

At 95% confidence, α_1-α/2_ = 1.96;

At 80% power, z_1-β_= 0.842;

P1= Estimated proportion of mothers without GDM (13%);

P2= Estimated proportion of mothers mothers with GDM (23%);

P1-P2= Effect size (10%);

P=(P_1_+P_2_)/2

The minimum required sample size is 231. Allowing for 10% attrition, the sample size will be adjusted upwards to 254 with each group having a sample of 127 respondents. Six maternity facilities were selected using simple random sampling method to ensure all facilities had an equal chance of inclusion as a study site given a population of 97 maternity facilities from Nairobi County Master Facility List (MFL) spread within six sub counties of Nairobi County. Facilities codes were recorded on sheets of papers, folded carefully, mixed in a container and hand picked randomly from the container.

Each code was then matched with the MFL and a total of six (6) had a chance of representation in this particular study. All women who attended clinic during the study period and had met inclusion criteria were purposively allowed to be part of study subjects until the suitable sample size was reached. A structured interviewer administered questionnaire was used to collect data and oral glucose intolerance test was done to ascertain GDM status of mothers selected to participate in the study

**Data management and analysis**: Filled questionnaires were checked for completeness thereafter the code data was entered into excel sheet which was exported for analysis onto stata version 13. Risk factors were analysed as odds ratio, univariate logistic regression and multilevel logistic regression to account for confounding variable. Background information of respondents was presented as frequency and percentages.

## Results

Results on demographic characteristics of respondents were presented in descriptive statistics. Table 1 indicates that out of 238 respondents 172 (72.27%) had no GDM condition while 66 (27.73%) had GDM condition. Most mothers 112 (47.86%) had BMI above normal (Overweight and Obese), 108 (46.15%) had normal weight and few 14 (5.98%) were underweight.

**Table 1:** Descriptive statistics on background information

Variable ( Respondents characteristics)	Frequency (n)	Percentage (%)
GDM mothers		
Yes	66	27.73
No	172	72.27
Diabetic history		
Yes	102	47.89
No	111	52.11

Diabetes history in the family was highly observed at 38 (62.67%) among mothers with GDM condition while no history of diabetic condition in the family was a common observation at 88 (57.89%) among non-GDM mothers while high blood pressure condition was common 23 (38.89%) among GDM mothers compared to 27 (18.75%) among non-GDM mothers (Table 2). The mean age for mothers with GDM was high with a mean of 33.06 (95% C.I: 31.59-34.52) compared to a mean of 27.9 (95% C.I: 27.01-28.78) for non- GDM mothers. Complimentarily, weight before pregnancy was high with mean of 74.04 (95% C.I: 70.82-77.30) among mothers with GDM compared to mean of 60.27 (95% C.I:58.59-61.96) among non-GDM mothers (Table 3).

**Table 2:** Distribution of independent variables between GDM mothers and Non-GDM mothers

Variable	GDM mothers (n=66)		Non GDM mothers (n=172)	
	Frequency	Percentage	Frequency	percentage
**Diabetic history**				
Yes	38	62.3	64	42.11
No	23	37.7	88	57.89
**Blood pressure**				
Normal	37	61.67	110	76.39
High	23	38.33	27	18.75
Low	0	0	7	4.86

**Table 3:** Mean distribution of age and weight before pregnancy between mothers GDM and non-GDM condition

	GDM mothers (n=66)		Non-GDM mothers (n=172)	
Mean age	33.06	(31.59-34.52)	27.9	(27.01-28.78)
Mean Weight before pregnancy	74.04	(70.82-77.30)	60.27	(58.59-61.96)

**Figure 1:**
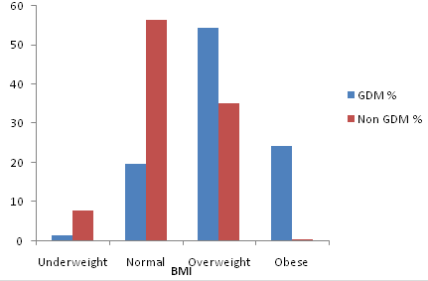
Distribution of BMI among GDM and non-GDM mothers

Results for BMI among mothers determined by weight before pregnancy indicates that mothers with GDM had above normal BMI (overweight and obese) at 54.55% and 24.24% respectively compared to non-GDM with 35.12% and 0.6% in the category of overweight and obese respectively (Figure 1). To further establish the relationship between gestational diabetes and individual biological characteristics, logistic regression analysis was done with results indicating that Mothers who had diabetic history in the family had twice the risk of developing GDM (OR= 2.27; 95% C.I: 1.23-4.17) compared to those who did not observe diabetic history in the family. Obese BMI status had positive association with GDM, findings indicates that obese mothers had two times increased risk of developing GDM condition (OR= 2.08; 95% C.I: 11.83-3656.7). Age and weight of the mother before pregnancy similarly had positive association with GDM (OR = 1.15; 95 % C.I:1.089-1.206) and (OR =1.10; 95 % C.I:1.07-1.13) respectively (Table 4).

**Table 4:** Univariate logistic regression model for the analysis of factors associated with development of gestational diabetes among mothers attending ANC in Nairobi County

Variable (n = 238)	OR	95%CI	P-value
Diabetic history			
No diabetic history (Reference)	1		
Had diabetic history*	2.27	(1.23- 4.17)	0.008
BMI before pregnancy			
Under weight (Reference)	1		
Normal	1.78	(0.214-14.74)	0.593
Overweight	7.93	(0.99-63.22)	0.051
Obese*	208	(11.83-3656.7)	0.000
Age of respondent*	1.15	(1.089-1.206)	0.000
Weight before pregnancy*	1.10	(1.07-1.13)	0.000

* **Significant at 5% significance level**

After adjusting for age confounder, GDM risk remains significant in age and weight (OR = 1.098; 95% C.I: 1.089 -1.206) and (OR = 1.05; 95% C.I:1.003 -1.093) respectively, although slightly reduced in magnitude where age has 9.8 % risk and weight gave 5% increased risk to the development of GDM, while diabetic history and BMI were not significant Table 5.

**Table 5:** Multivariate logistic regression model for the analysis of factors associated with development of GDM among mothers attending ANC

Variable (n = 238)	OR	95%CI	P-value
Diabetic history			
No diabetic history (Reference)	1		
Had diabetic history	1.19	(0.53-2.71)	0.671
BMI before pregnancy			
Under weight (Reference)	1		
Normal	1.20	(0.129-11.22)	0.870
Overweight	1.69		0.647
Obese	18.69	(0.176-16.37) (0.766456.38)	0.072
Age of respondent*	1.098	(1.089-1.206)	0.000
Weight before pregnancy*	1.05	(1.003-1.093)	0.036

* **Significant at 5% significance level**

## Discussion

The analyses in this study suggest that individual biological factors such age is independently associated with GDM, including weight of the mother before pregnancy and history of diabetes among family members. Further, the study suggests that GDM is common among women who are advanced in age, mothers with GDM had a mean age of 33.06 years while non-GDM mothers had a mean age of 27.9 years. These findings are consistent with evidence that any women above age of 25 years has increase risk of GDM. High BMI which corresponds with high weight was associated with GDM condition. This study indicates that nearly more than half (47.86%) of the study population is considered either overweight or obese. According to Macaulay et. al (2014), diabetes and other NCDs such high blood pressure among others are largely associated with sedentary life style as populations shift from rural way of life to urban life lifestyle to a more industrial urbanized way of life and hence obese condition among population. Obesity condition has serious health consequences related to NCDs including diabetes and complications during pregnancy and subsequent generations. Mothers who had a close family members sibling or parent with diabetes are likely to develop gestational diabetes this is evident by the fact that mothers with GDM condition reported 62.3% observation in diabetes in family tree while mothers with non GDM condition reported low 42.11% observation in family members who had diabetes.

## Conclusion

Gestational diabetes cases is relatively high in the population, lifestyle including other individual biological characteristics such as advanced age in pregnancy, high weight and diabetic history in family are determining factors for development of diabetes among pregnant women.

### What is known about this topic

- Studies in the US and ASIA have been able to establish the prévalence of diabètes amongits population;
- Maternal factors including medical condition, weight, age and nutrition during development of fetus is a determinant of health and disease susceptibility in a population;
- Sedentary life that is increasing as population embrase urban life style is a keycontributor of increasingprevalence of GDM.

### What this study adds

- Little is known about diabetes in Africaowing to the factthat no much research has been done on non-communicable diseases, hence this study sought to establish contextual maternal factors that determine population health;
- The studyestablished incidence of GDM amongurban population living in Nairobi;
- The studyrecommends for inclusion of diabetestesting and management as package in Maternal and Child Health services during the visits to antenatal clinics providing information on how to manipulate prevailing condition to the development of GDM.
